# Evaluation of a newly developed first aid training programme adapted for older people

**DOI:** 10.1186/s12873-023-00907-6

**Published:** 2023-11-10

**Authors:** Eva Dolenc Šparovec, Damjan Slabe, Ivan Eržen, Uroš Kovačič

**Affiliations:** Faculty of Health Sciences, Sanitary Engineering Department, Public Health Division, University of Ljubljana, Zdravstvena pot 5, Ljubljana, 1000 Slovenia

**Keywords:** Basic life support, Elderly, Hypoglycaemia, Stroke, Out-of-hospital Cardiac Arrest

## Abstract

**Background:**

Older people need to acquire knowledge and skills at first aid (FA) training tailored to them. Our research aimed to evaluate an FA training programme adapted for older people. We assumed that satisfaction with FA training, as well as knowledge of FA, would be higher among older people who received training according to an adapted programme compared to those who received training according to the existing programme for the general public.

**Methods:**

We trained older people according to the existing FA programme for the general public and according to a new FA training programme adapted for older people. The new training program is shorter and focuses on FA contents that are more relevant for older people. We evaluated participants with a general assessment questionnaire (consisting of items regarding satisfaction, comprehensibility, length, and physical difficulty), a test on theoretical FA knowledge, and a test on practical cardiopulmonary resuscitation (CPR) knowledge. To ensure the homogeneity of the groups and to verify the impact on the results of the test of practical CPR knowledge, we also tested the participants regarding their psychophysical capabilities.

**Results:**

A total of 120 people completed the free FA training sessions. The general assessment questionnaire score of participants who were trained based on the new FA training program was 19.3 (out of 20), which was statistically significantly (p < 0.05) higher than that of those trained based on the old program (general assessment score of 17.1). Participants who were trained based on the new program scored an average of 8.6 points on the theoretical FA knowledge test, while those who were trained based on the old program scored an average of 7.1 points, which was statistically significantly (p < 0.05) lower. In both programs, the same average scores (7.5 out of 10 points) on the practical CPR knowledge test was achieved. However, participants who participated in the FA course adapted for the older people gained practical CPR knowledge in a shorter time. Older people with a greater psychophysical capacity were more successful in performing CPR, regardless of which FA training programme they received.

**Conclusions:**

The effectiveness of FA training is greater if older people are trained in accordance with a targeted programme adapted to the psychophysical limitations of the older people.

**Supplementary Information:**

The online version contains supplementary material available at 10.1186/s12873-023-00907-6.

## Background

When a disease or injury develops, appropriate bystander first aid (FA) measures can improve the outcomes of individuals with out-of-hospital cardiac arrest [1–4] or trauma [5] victims. Based on FA guidelines [6–8], there are many organisations that promote and implement FA courses around the world. Media campaigns, mass training sessions, focused refreshers, resuscitation education for adults, and the education of school children are effective and long-lasting ways to increase bystander efforts [9].

In contrast, Birkun et al. [10], in their scoping review, warned that nothing is known about existing community resuscitation education coverage for most countries of the world; moreover, for most surveys, the findings are not generalisable to a whole country’s population. Some countries reveal, in different ways, an increase in cardiopulmonary resuscitation (CPR) training prevalence over time. These studies were carried out in Australia [11], China [12, 13], Japan [14, 15] and South Korea [16]. Nevertheless, there is an apparent need to improve public awareness and education on resuscitation internationally [10]. The findings from another scoping review [17] identify high levels of perceived knowledge, confidence, and willingness to help but also point to low uptake levels, low tested skill-specific knowledge, and barriers to learning FA and helping, indicating that the FA training landscape needs improvement. Oliver [18] explained that FA education has traditionally fallen into the gap between emergency medicine and public health. One of the reasons is that it remains behind the curve in modern educational approaches. Traditionally, it has been about teaching complex medical interventions to people with no medical background in a face-to-face setting. It is only very recently that providers have simplified language and steps, shortened courses, introduced digital learning elements, and recognised that giving people the confidence to act is just as important as learning the skills. Oliver [18] emphasises that, in the field of FA education, more evidence is needed on how to reach different populations in different cultures and contexts and how to bridge the gap between emergency response and FA as a public health tool.

In Slovenia and elsewhere in the world, FA training programmes do not sufficiently consider the capabilities and needs of individual population groups, among whom older people stand out [19]. In contrast to younger people, a smaller proportion of older people receive FA training [20]. Morin and colleagues [21] stated that in coming years, France intends to provide a ‘Senior Force Against Cardiac Arrest’ in addition to youth training at schools. There are many reasons to believe that policies of basic life support training must be intensified for this population. The idea is not new; back in 1989, Pane and Salnes [22] researched the targeted recruitment of older citizens and cardiac patients, focusing on a mass CPR training course. Thus far, there have been some successful FA courses for older people in various countries [23–25]. Vaillancourt et al. [26] identified key facilitators and barriers for CPR training and performance in a purposive sample of laypersons aged 55 years and older. Older people are a very specific group in terms of experience, training, and psychophysical capabilities. In other studies [19, 27], the authors of the present paper revealed that older individuals are very differently motivated to participate in FA training due to heterogeneity in their psychophysical capabilities. They need and want to obtain additional knowledge about FA and health protection for which any psychophysical limitations are not as relevant as when learning CPR. They want to learn how to recognise emergency situations and call emergency services with the use of modern technology. In addition to CPR without rescue breaths, they also want to learn about topics related to the treatment of injuries. They want this training to be provided as short course, adjusted to their varied psychophysical capabilities. One of the key characteristics of older people seems to be their great diversity compared to the younger population [28]. Krammel et al. [29] suggested that specifically tailored programmes to increase awareness and willingness in the older adult community need to be considered for future educational interventions. Dolenc et al. [30] determined that older individuals need to acquire knowledge and skills through FA training tailored to them.

Our research aimed to evaluate a tailored FA training programme adapted for older people. We assumed that satisfaction with FA training would be higher among older people who receive training according to an adapted programme compared with those who receive training based on the existing programme for the general public, as well as theoretical knowledge of FA and practical knowledge of CPR after FA training.

## Methods

We trained older people according to the existing four-and-a-half-hour FA programme for the general public (control group) or according to the new FA training programme adapted for older people (experimental group). The study took place from February 2022 to June 2023. The training sessions were conducted in different places in Slovenia (city and/or countryside).

### Study design

**Fig. 1:**
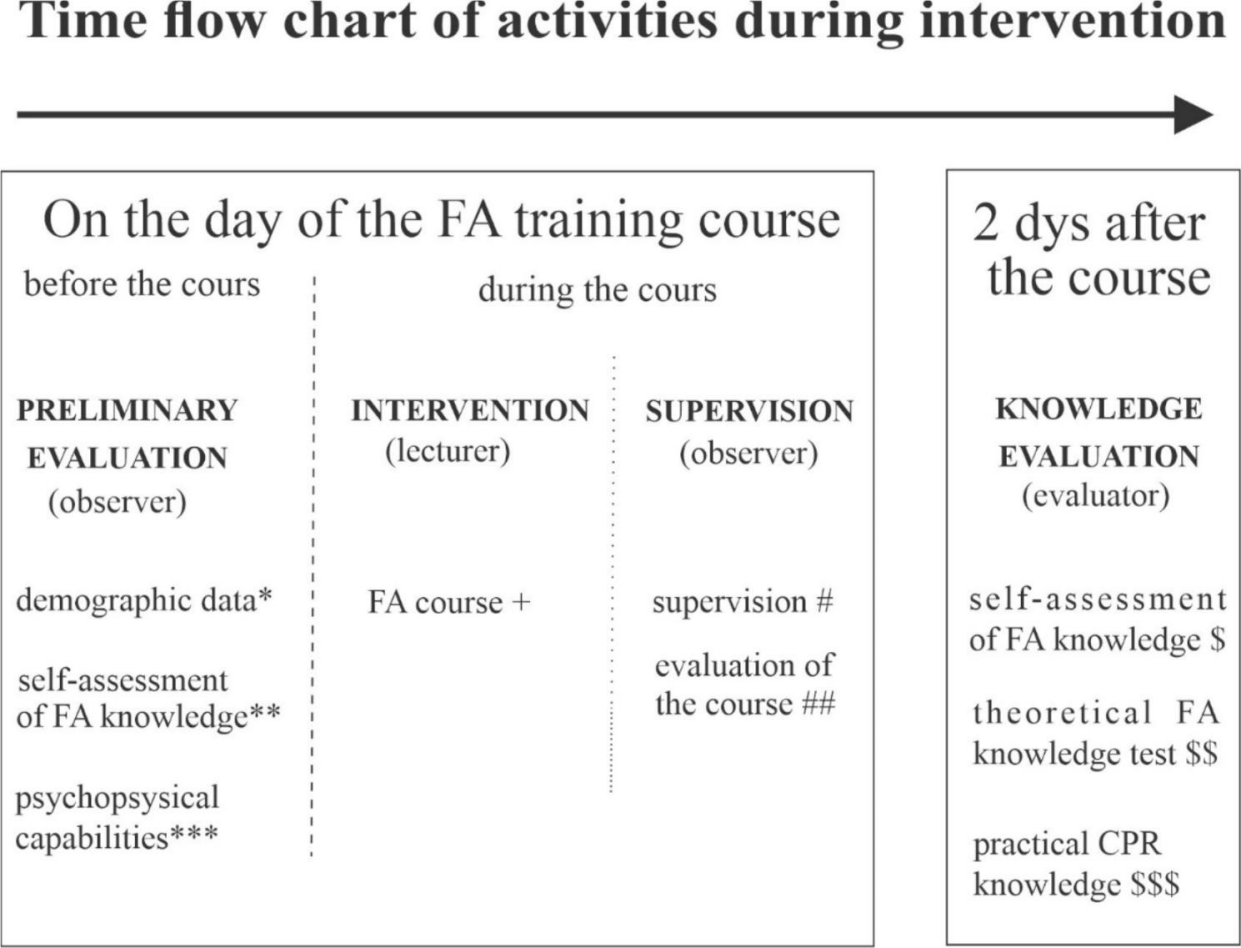
Time flow chart of activities before and during the course implementation and knowledge testing two days after the course. * & ** see Appendix A1; ***The Montreal Cognitive Assessment [27] & the physical capability test battery (see Appendix C). + two different courses were performed (old version and newly adapted to older participants; for details see Appendix B). # recording the details and special statements of participants (personal notes of observations); ## see Appendix A2. $ see Appendix D; $$ In theoretical knowledge test we asked about (1) recognition of symptoms and FA action in hypoglycemia, stroke, myocardial infarction, (2) FA measures in a person without signs of life, (3) FA action in the case of a hip fracture, (4) alcohol use and home-made ointments in FA measures (for details see Appendix D); $$$ Resusci Anne QCPR resuscitation model Skill Guide with the free mobile app QCPR Learner

We conducted a randomised double-blinded controlled trial. Participants were volunteers who attended FA training sessions for older people. We invited potential participants with promotional leaflets (posters), which we sent to various associations and posted in institutions where the older people lingered more often (University for the Third Life Period, gyms where the older people go, Red Cross centres, day care centres for older people). Individuals who were interested in the FA course signed up for a specific appointment. We held the courses when we had enough applicants. We did not know their demographic characteristics in advance, except their age and sex. The inclusion criterion was an age of 60 years or older. We randomly assigned the applicants to the control or experimental group except for the last two FA training sessions. After conducting 13 courses, we checked the demographic characteristics of the two groups and then collected additional applications. Thereafter, we conducted two additional courses where 18 participants were nonrandomly assigned in order to balance the groups in terms of gender and age. To ensure the homogeneity of the groups and to verify the impact on the results of the test of practical CPR knowledge, we also tested the psychophysical capabilities of all participants before the FA course started. The groups, were balanced in this regard, even if we did not analyse the data before the completed course. The data collection and course delivery process are described in Fig. 1.

The coauthor of the study organised all FA training sessions (posting of posters, collection of applications, timetable, notification of participants, communication with the teacher, observer and evaluator, preparation of aids, printing of questionnaires, etc.). The intervention consisted of FA training sessions conducted under two different programs (one for the experimental and other for the control group) that the authors of the study randomly communicated to the lecturer a few hours before the start of the lecture. The lecturer was qualified to implement both programs. To ensure homogeneity in training, all FA training sessions were led by the same licenced FA lecturer. He was licenced as an FA instructor and had a university medical degree. Sessions were supervised by a person who observed the lectures and performed data collection, recorded the details and special statements of the participants. Participants were not aware that we were comparing two courses with different contents. Before the course, participants completed questionnaires about demographic data and a self-assessment of their FA knowledge (see Appendix A1). The training programme was evaluated with a general assessment questionnaire (consisting of items regarding satisfaction, comprehensibility, length, physical difficulty; see Appendix A2) immediately after the course was completed. Two days after the training the theoretical FA (see Appendix D) and practical CPR knowledge of participants was tested by a third person (evaluator), who was not aware to which group the participant belonged to. The theoretical FA and practical CPR knowledge took place over two days because we wanted to prevent the meeting from being too long (to prevent overloading the older adults). At that time, the participants also self-assessed their FA knowledge after the training. All tests were coded so that participants remained anonymous. The lecturer, observer and evaluator were independent (not familiar with the exact purpose of the study) and not part of the group of authors of the study. Just like the lecturer, the observer and evaluator were also licenced FA instructors and had a university medical education.

### First aid training programme

In our previous research [31], we found that no programme for an FA course adapted for older people has been formally adopted in Europe. Based on our previous research and the current guidelines [6, 7, 32] we prepared a new FA programme for older people, which we evaluated in this study. The curriculum is shown in Supplementary Appendix B. For comparison, we used the existing FA training programme for the general public (see Appendix B) since we did not find a formally adopted tailored FA programme for older people. The main difference between the old (control program) and the newly adapted program is the duration. The adapted new program is short (it lasts for 155 min), while the old program is 270 min long. The new program has a shorter introductory theoretical part, some simplifications in the CPR procedures and changes in the injury chapter. When designing the new program for older people, we did not want to remove important topics of FA, and we tried to keep only the key information for understanding and providing FA in real situations. Therefore, in the new programme we excluded the topics of triage, infant and child CPR, rescue breaths, CPR after drowning person and acute poisoning. In the accident approach and procedures, we emphasised on calling an ambulance. In the field of sudden injuries, we focused on FA contents that are more relevant for older people (injures from falls e.g., hip injury). Both training programmes contained theoretical and practical components. In the new program, the practical exercises are significantly shorter for ​​injuries, but not so much in CPR procedures.

### Participants

One hundred thirty-one (131) people responded to the invitation to participate in free FA training sessions. The sessions were intended for people over the age of 60 years, and participation was voluntary. We conducted 15 sessions including between 6 and 10 participants. All participants signed written informed consent forms. Individuals who were immobile, blind, or deaf, or had similar health limitations did not participate in the course. Persons who wished to withdraw from the survey during the training had the opportunity to leave any time. Eleven (11) participants from the control group left the untailored training before the end of the program. We did not obtain any information from them, except the reason why they were leaving the course. Regarding the reasons, they stated that the training was too long, or they had other obligations that day. As we could not obtain the necessary data from them, we did not include them in the final analysis of the results. The control and experimental groups included 66 and 54 participants, respectively.

### Data collection instruments

Before the training demographic data, general characteristics (see Appendix A1) and data on self-assessed FA and CPR knowledge (scored on a scale from 1 to 5; see Appendix A1) were obtained from the participants. The training was evaluated with a general assessment questionnaire (see Appendix A2), which the participants completed immediately after the training. It consisted of satisfaction, comprehensibility length and physical difficulty assessments (scored on a scale from 1 to 5). We also assessed the psychophysical status of the participants. The Montreal Cognitive Assessment [33] test was used to determine the mental capacity of the participants. Prior to use, we completed a training module and obtained a certificate [33] that allows the researcher to use the Montreal Cognitive Assessment test. It is a cognitive screening test designed to assist health professionals in the detection of mild cognitive impairment and Alzheimer’s disease. A 30-item test enables can determine how well a person’s thinking capabilities are functioning. The test checks language, memory, visual and spatial thinking, reasoning, and orientation skills. The physical capability test battery (see Appendix C) was adapted according to the Senior Fitness Test [34], the Balance Scale [35] and the Four Square Step Test [36]. We wanted to use tests that measure the physical capabilities required to perform CPR (biceps test, getting up from a chair, balance tests, picking up an object from the floor, etc.). The set of five tests showed us a rough picture of the physical performance of the participants.

Two days after the training self-assessment of FA and CPR knowledge were completed again (in the analysis, we used the difference in the self-assessment of knowledge before and after the training, scored on a scale from 1 to 5). All participants also completed the test about their theoretical knowledge of FA (see Appendix D). The test contained questions to which all training participants in both programs received answers during the training. All 10 questions were related to the emergency conditions and injuries that often occur in old age. Participants were coded and the evaluator was not aware to which group (control or experimental) a participant belonged. To evaluate CPR performance, we used the Resusci Anne QCPR resuscitation model Skill Guide (Laerdal, Norway) with the free mobile app QCPR Learner (Laerdal Medical, United States). The Resusci Anne model simulates an adult with average physiological measurements and is designed for realistic training in basic CPR techniques. Every older person included in the study was asked to perform CPR without rescue breaths (as previously taught in FA training) on the adult model for one minute. We chose chest compression only CPR even though the guidelines [37] recommend 30 compressions and 2 breaths for bystanders who are trained, able, and willing to give rescue breaths. However, if bystanders are not trained or are unable to give rescue breaths, they are also allowed to give chest-compression only CPR. Research on older people shows that they find it more difficult [38] or are unwilling [19] to give rescue breaths which is the reason why we choose this. After the performance of CPR, we obtained results using the mentioned mobile app. The programme recorded hand position, chest compression depth, release, frequency, and rate. The parameters that were considered correct were based on the European Resuscitation Council guidelines [7] for adults: a rate of at least 100–120 compressions per minute with a compression depth of at least 5 cm at the lower third of the sternum. We used function compressions only. Based on the recorded parameters, the mobile application provided overall score results [39].

### Data analysis

Statistical analysis was conducted using SPSS 22.0 (Statistics Package for Social Sciences, Chicago, IL, USA) software. The difference in general characteristics between the experimental (E) and control (C) group was compared using the χ^2^ test. Differences between groups regarding the number of points achieved on psychophysical performance tests, knowledge tests and general assessment of FA course were compared using Mann-Whitney U tests. We performed preliminary analyses for normality. Given that the distribution was not normal, we performed a nonparametric test. Regardless, we presented the results for a more straightforward interpretation and comprehensibility of the averages and standard deviations compared to the ranks. With regression analysis, we determined the correlation among the variable quality of CPR performance (number of points on the CPR practical knowledge test) and psychophysical capabilities (The Montreal Cognitive Assessment and physical testing) of the respondents. We performed preliminary analyses for normality. Given that the distribution was not normal, we used Spearman’s rho. A p-value of < 0.05 was statistically significant.

## Results

### General characteristics of participants

Most participants were women with higher education. 65% of all participants were between 60 and 70 years old and for more than half of them, more than 10 years had passed since their last FA training. In both groups, participants scored an average of 26 points on mental performance tests and approximately 13 points on physical tests. Slightly less than half of the participants from the control and experimental groups stated that they had some health problem that hindered them on a daily basis (osteo-muscular, cardio-respiratory, or neurological diseases). Nevertheless, a relatively high percentage of participants were active (recreational sports, working in the garden, mushroom picking) and approximately half of them also looked after their grandchildren (Table 1). We found no statistically significant difference (p > 0.05) between the control and experimental groups in the general characteristics of the participants (Table 1).

**Table 1:** General characteristics of the participants (N = 120); comparison between the experimental and control groups

Category		E (n = 66)	C (n = 54)	χ2 or U	p*
		% (n) or x̄ (σ)	% (n) or (σ)		
SEX	female	82% (54)	76% (41)	0.501	0.625
	male	18% (12)	24% (13)		
AGE (years)	60–70	70% (46)	60% (32)	1.481	0.477
	71–80	18% (12)	25% (14)		
	> 80	12% (8)	15% (8)		
YEARS FROM LAST FA COURSE	< 5	17% (11)	28% (15)	3.181	0.582
	5–10	23% (15)	22% (12)		
	> 10	55% (36)	44% (24)		
	never	7% (4)	6% (3)		
EDUCATION^#^	primary	12% (8)	20% (11)	2.005	0.571
	vocational	14% (9)	17% (9)		
	academic	35% (23)	30% (16)		
	higher	39% (26)	33% (18)		
RESIDENCE	the countryside	44% (29)	50% (27)	5.399	0.067
	small urban centres	42% (28)	48% (26)		
	larger cities	14% (9)	2% (1)		
MENTAL CAPABILITY (max 30 points)		25.98 (2.2)	25.81 (2.68)	1760.5	0.908
PHYSICAL CAPABILITY (max 16 points)		13.18 (2.9)	13.02 (2.5)	1625.5	0.404
I WAS (AM) HEALTH CARE PROVIDER.		12% (8)	8% (4)	0.733	0.294
I HAVE A SICK FRIEND, A RELATIVE.		56% (37)	70% (38)	2.595	0.077
I HAVE A HEALTH PROBLEM.		41% (27)	48% (26)	0.631	0.271
I TAKE CARE OF GRANDCHILDREN.		52% (34)	46% (25)	1.454	0.483
I AM ACTIVE.		92% (61)	83% (45)	2.382	0.105

E: experimental group; C: control group; mean; x̄: mean; σ: standard deviation; * p < 0.05 – significant difference between E and C group; ^#^education: primary school, secondary school – vocational. secondary school – academic, higher – college, university

### Effectiveness and efficiency of FA training

There were differences between the experimental and control groups on the general assessment of the FA training and theoretical FA knowledge test, for which the experimental group achieved a significantly (p < 0.05) higher number of points. As a result, the experimental group also achieved a significantly higher (p < 0.05) total number of points (37.9 points on average) compared to the control group (34.1 points on average). Regardless of the type of training, the experimental and control groups scored approximately 7.5 points on the practical CPR knowledge test and the difference between the groups was not statistically significant. (Table 2)

**Table 2:** Comparison of differences in the effectiveness and efficiency of FA training between the experimental and control groups

Effectiveness and efficiency criteria	E (n = 66) x̄ (σ)	C (n = 54) x̄ (σ)	U	P
GENERAL ASSESSMENT OF FA TRAINING (max 20 points)	19.3 (1.2)	17.1 (2.9)	992.0	< 0.001*
CHANGE IN FA AND CPR KNOWLEDGE SELF-ASSESSMENT (max 8 points)	2.6 (1.4)	2.5 (1.5)	1684.0	0.591
THEORETICAL FA KNOWLEDGE TEST (max 10 points)	8.6 (1.2)	7.1 (1.5)	808.0	< 0.001*
PRACTICAL CPR KNOWLEDGE TEST (max 10 points)	7.5 (3.5)	7.5 (3.6)	1636.0	0.472
TOTAL NUMBER OF ALL POINTS (max 48 points)	37.9 (4.6)	34.1 (5.4)	897.5	< 0.001*

E: experimental group; C: control group; mean; x̄: mean; σ: standard deviation; * p < 0.05 – significant difference between E and C group

### General assessment of FA training

The general assessment of the FA training sessions consists of four different criteria. There was a statistically significant difference (p < 0.05) between the experimental and control groups in the assessment of the satisfaction with and length of training sessions (Table 3). The poorer assessment of the control groups regarding the length of FA training stood out. For most participants from both groups, the FA training sessions were completely understandable. Both groups also assessed the physical difficulty of the training as appropriate or not demanding.

**Table 3:** Group comparison of differences within the general assessment criteria

General assessment criteria	E (n = 66) x̄ (σ)	C (n = 54) x̄ (σ)	U	p
SATISFACTION (max 5 points)	4.8 (0.5)	4.5 (0.7)	1489.0	0.046*
COMPREHENSIBILITY (max 5 points)	4.8 (0.4)	4.7 (0.5)	1552.0	0.085
LENGTH (max 5 points)	4.4 (1.5)	3.6 (1.9)	1448.5	0.019*
PHYSICAL DIFFICULTY (max 5 points)	4.9 (0.3)	4.8 (0.5)	1601.0	0.123

E: experimental group; C: control group; mean; x̄: mean; σ: standard deviation; * p < 0.05 – significant difference between E and C group; assessed on a scale of 1 to 5 where 1 means a poor grade and 5 means an excellent grade

### Influence of psychophysical capabilities on the quality of CPR performance and on the theoretical FA knowledge

We tested the correlation among the variables of psychophysical capabilities (the Montreal Cognitive Assessment and physical testing) and quality of CPR performance (from the number of points achieved on the practical CPR knowledge test; the maximum total score was 10) and on the theoretical FA knowledge (from the number of points achieved on the theoretical FA knowledge test; the maximum total score was 10) with regression analysis. The correlation between mental (psycho) capabilities and CPR knowledge was positive (correlation strength 0.245; which means low or weak connection), and the correlation was statistically significant (p < 0.05; p = 0.007). Correlation between the Montreal Cognitive Assessment score and FA knowledge test score was also statistically significant (p < 0.05; p = 0,038), positive and low (correlation strength 0.190). The number of points scored on the CPR knowledge test was also correlated with the physical capabilities of the respondents (correlation strength 0.622, which means medium or moderate connection), and the correlation was statistically significant (p < 0.001). Correlation between the number of points achieved on the FA knowledge test and physical capabilities was not statistically significant (p > 0.05; p = 0.225). There were no differences between the experimental and control groups in regard to the correlations between psychophysical capabilities and knowledge.

## Discussion

In this study we evaluated the effectiveness and efficiency of newly developed FA educational programme tailored for older people in Slovenia. To the best of our knowledge, this is one of the first attempts to compare different educational FA programmes for older people. We found that the effectiveness and efficiency of FA training were greater if older people were trained in accordance with a targeted programme for the population. Satisfaction with FA training, as well as the theoretical knowledge of FA, were higher among older people who received FA training according to an adapted programme compared to the existing programme for the general public. Older people who participated in the FA course adapted for older people gained practical CPR knowledge in a shorter time. Given that older people want shorter courses [19], this could motivate them to participate. With a shorter course, we can also effectively train more people in less time. Heterogeneity in the psychophysical capabilities of older people affected CPR performance success, regardless of which FA programme they received. Older people with greater psychophysical capacity were more successful in performing CPR. An obstacle to the acquisition of theoretical FA knowledge in older people is cognitive decline, not physical ability. The psychophysical characteristics of older people also affected their motivation to participate in FA training. As expected, the training was attended by those with more preserved capabilities.

In general, older people who chose to receive our FA training were mostly active, higher educated women up to 70 years of age with good psychophysical capabilities. According to related research FA training also tends to be undertaken by individuals who are younger, are male, or have a higher level of education [17, 40, 41]. Slightly less than half of the respondents stated that they had some health problems. Many more of them had ill relatives or friends. Nevertheless, they were active and approximately half looked after their grandchildren. In general, after children, older people represent the second most vulnerable group of the population for injuries and sudden illnesses [42]. The average number of points received by the participants in our FA training sessions (13 out of 16 points on the physical performance test and 26 out of 30 points on the mental test) may also indicate a decrease in psychophysical capacities and thus an increase in vulnerability. Nevertheless, psychophysical decline is a well-known fact [43–45]. Physical limitations may only be an obstacle in the practical implementation of procedures such as CPR, but not in the acquisition of theoretical FA knowledge.

Older people are at risk for out-of-hospital cardiac arrest or trauma; at the same time, they are potential FA providers for individuals with such complications. One challenge is the motivation of the older to participate in FA courses, which varies greatly [24, 25]. When organising an FA course in Slovenia, special attention should be devoted to the motivation of men and those over the age of 80 years, for whom it would be necessary to identify for potential alternative ways of providing FA education, as this group is the most vulnerable among the older population [31]. When planning courses, it is necessary to consider that longer and content-intensive courses are too demanding for older people. We found that they leave such courses prematurely or do not acquire as much FA knowledge as in the target course.

After the performance and final evaluations of FA training sessions, we found significant differences in the satisfaction of participants who attended a training session tailored to older people. The results showed that the participants rated both versions of the training as very good to excellent regarding most indicators (except for the length of the untailored training sessions). According to our observations, high grades were attributed to the general usefulness of the FA topic. Participants were also satisfied with socialising with peers while acquiring useful knowledge; they mentioned this several times (observer’s note). Participants from the experimental and control groups showed much interest in the content, asked questions, performed practical exercises, and shared life experiences (personal notes of the observer and trainer). A similar positive experience was described in the evaluation of FA training for older people conducted in other studies [23–25].

An important difference between the experimental and control groups was the assessment of satisfaction with the length of training sessions. Due to that significant difference in favour of the tailored training programme, we must also consider the fact that 11 participants left the old (longer) FA training programme prematurely. Most of them dropped out because of the length of this programme. Data about the dropouts confirms that the old program is too long and too demanding for older people. Towards the end of the programme, we also observed signs of impatience and fatigue: participants engaged with the training less and looked at the clock more (observer’s notes). We confirm the findings [46, 47] that FA training courses should be tailored to the target audience and kept as simple as possible; course organisers have to plan their courses in a flexible manner, allowing for a shorter duration for target groups with different backgrounds and more hands-on time for laypersons. As a critical difference between the two types of courses, we considered the better knowledge of the experimental group participants who participated in the adapted course. Participants who participated in the new FA course adapted for older people gained more FA knowledge in a shorter time.

Regardless of the type of FA training, the experimental and control groups scored approximately 7.5 points on the practical CPR knowledge test. As the difference between the groups was not statistically significant, we cannot conclude that the older people who participated in the FA course adapted for older people gained more practical CPR knowledge. Nevertheless, they gained CPR knowledge in a shorter time, which additionally confirms the noninferiority of the new FA course. We also found that CPR performance is influenced by the psychophysical capabilities of older people, who cannot be taught through training. Accordingly, Leary et al. [48] confirmed that women and the older people perform shallower chest compressions on average. Similar findings were also confirmed by other researchers [49–52]. Nonetheless, we found that older people with preserved physical performance were able to effectively perform CPR on the Resusci Anne device for one minute. As part of our study, participants in both courses performed chest compressions only. This is in accordance with related studies that showed that, after CPR training, older participants also performed effective resuscitation [24, 53]. It is also important to train people in the correct way, which leads to better CPR performance [54]. A shorter and tailored course can make this possible.

In summary, heterogeneity in the psychophysical capabilities of older people affects not only their motivation to participate in FA training but also their ability to perform CPR, which is usually part of any FA training programme. The FA training sessions that we evaluated in our research also included other FA topics, in addition to CPR. With such a programme, even older people with limited physical capabilities can acquire useful FA knowledge. At the same time, FA is also an act of solidarity and an expression of humane action towards other human beings, so older people should not be deprived of this knowledge.

### Limitations

The programme that we designed and evaluated was tested on a sample of Slovenian older people and can be locally adapted. It would make sense to evaluate such a programme in a different (cultural) environment. The study included older people who voluntarily participated in FA training. These were people with mostly preserved psychophysical capabilities, which excludes the possibility of representativeness of the sample to the entire older population in Slovenia. In addition to psychophysical capabilities, there are many other factors that can influence the obtained findings (social, economic and others), namely older people are a very heterogeneous population group. The final results of all trainings could be influenced by the lecturer, but an additional person (observer) took care of the control over consistency and the exact following of the set program. The lecturer only conducted the courses according to the program and lectured independently of program evaluation, which was anonymous and objectively performed by third person (evaluator). Due to limited capacity (time, aids), the instrument for measuring physical performance was adjusted. It would make sense to use a validated instrument and compare the results. When designing the test battery, we wanted to include measurements that could have the greatest impact on CPR performance. To obtain better insight into the connection between mental capacity and the ability to perform CPR, we should purposefully recruit people with poorer Montreal Cognitive Assessment results or more significant cognitive decline. Such people did not attend the FA training voluntarily. To increase the effectiveness of our study, it would be reasonable to follow up the participants at a later time frame to determine how they retained the acquired FA knowledge. In the future, it would also make sense to validate a questionnaire on the FA knowledge of older people.

## Conclusions

There is a need for older individuals to acquire knowledge and skills through tailored FA training. The programme must be adapted so that even older people with poorer psychophysical capabilities can acquire the necessary knowledge to manage their health conditions and the problems that old age brings. The psychophysical characteristics of older people also affect their motivation to participate in FA training. A tailored short-term FA course can potentially motivate people with reduced psychophysical capacity to participate. Heterogeneity in the psychophysical capabilities of the older people also affected their CPR performance, regardless of the FA programme in which they were trained. This is another reason why a traditional FA programme for the general adult public is not always suitable for the older population.

### Electronic supplementary material

Below is the link to the electronic supplementary material.

Supplementary Material 1

Supplementary Material 2

Supplementary Material 3

Supplementary Material 4

## Data Availability

The datasets generated and analysed during the current study are not publicly available but are available from the corresponding author on reasonable request.

## Abbreviations

FA: First aid
CPR: Cardiopulmonary resuscitation
Moca: Montreal Cognitive Assessment
SD: Standard deviation
E: Experimental group
C: Control group
