# Supplementary material for: Evaluation of a newly developed first aid training programme adapted for older people
Source: BMC Emerg Med. 2023 Nov 10;23:134. doi: 10.1186/s12873-023-00907-6 (PMC10636823; doi:10.1186/s12873-023-00907-6)
Supplement: Supplementary file 1 — Supplementary Material 1 [file 12873_2023_907_MOESM1_ESM.docx]

APPENDIX A1

**DEMOGRAPHIC DATA**

**Sex:**

- Female
- Male

**How old are you? _____________years**

**What is your education?**

- Primary
- Vocational
- Academic
- Higher

**Where do you live?**

● In a retirement home

● At home, in the countryside

● At home, in a small urban centres

● At home, in a larger city

**How many years has it been since you last had a first aid course?**

- I've never had first aid training
- < 5 years
- 5-10 years
- > 10 years

**Please rate your knowledge of BASIC RESUSCITATION PROCEDURES (1 means very poor knowledge, 5 means excellent knowledge).**

1 2 3 4 5

**Please rate your knowledge of OTHER FIRST AID THEMES (not RESUSCITATION) (e.g. bleeding, care of injuries, measures in case of complications of diabetes, stroke, heart attack...) (1 means very poor knowledge, 5 means excellent knowledge).**

1 2 3 4 5

**Please answer the following statements.**

**I was (am) health care provider.**

- Yes
- No

**I have a sick friend, a relative.**

- Yes
- No

**I take care of grandchildren.**

- Yes
- No

**I am active in my spare time.**

- Yes
- No

What activity do you do? ___________________

**In my daily life, I am hindered by a health problem in my daily activities.**

- Yes
- No

What is this health problem? _________________

APPENDIX A2

[**GENERAL ASSESSMENT QUESTIONNAIRE**](https://www.google.com/search?sca_esv=577441552&sxsrf=AM9HkKkG6pFzYrkYKCtivvI60ofPIWPzLQ:1698498866843&q=GENERAL+ASSESSMENT+QUESTIONNAIRE&spell=1&sa=X&ved=2ahUKEwjQsu-s6ZiCAxWm_bsIHawyCHEQkeECKAB6BAgJEAE)

**Please rate how satisfied you are with the first aid course (1 means poor rating, 5 means excellent rating).**

1. 2 3 4 5

**Please rate the comprehensibility of the content presented in the course (1 means difficult to understand, 5 means completely understandable).**

1. 2 3 4 5

**Please rate the length of the course**

**(1 means unsuitable length, 5 means perfectly suitable length).**

1 2 3 4 5

**Why you rated 1, 2 or 3:**

the course is too short or the course is too long

**Please rate first aid course in terms of physical difficulty**

**(1 means too demanding, 5 means completely NOT demanding).**

1. 2 3 4 5

**Please rate your knowledge of BASIC RESUSCITATION PROCEDURES (1 means very poor knowledge, 5 means excellent knowledge).**

1 2 3 4 5

**Please rate your knowledge of OTHER FIRST AID THEMES (not RESUSCITATION) (e.g. bleeding, care of injuries, measures in case of complications of diabetes, stroke, heart attack...) (1 means very poor knowledge, 5 means excellent knowledge).**

1 2 3 4 5
