# Supplementary material for: Evaluation of a newly developed first aid training programme adapted for older people
Source: BMC Emerg Med. 2023 Nov 10;23:134. doi: 10.1186/s12873-023-00907-6 (PMC10636823; doi:10.1186/s12873-023-00907-6)
Supplement: Supplementary file 2 — Supplementary Material 2 [file 12873_2023_907_MOESM2_ESM.docx]

APPENDIX B

**TAILORED FIRST AID TRAINING PROGRAMME, ADAPTED FOR ELDERLY PEOPLE (for experimental group)**

| **TOPICS** | **TOTAL TIME** | **THEORY (minutes)** | **PRACTICAL EXERCISE (minutes)** |
| --- | --- | --- | --- |
| 1. definition of first aid (FA), importance of FA, moral and legal aspects of FA, objectives of FA | 5 min | 5 min | / |
| 1. approach to the victim - safety, responsiveness, airway, breathing | 10 min | 10 min | / |
| 1. call an ambulance | 10 min | 5 min | 5 min |
| 1. position for unconscious victim | 20 min | 10 min | 10 min |
| 1. cardiopulmonary resuscitation (CPR) (of adult) | 30 min | 15 min | 15 min |
| 1. use of an automatic external defibrillator (AED) | 15 min | 5 min | 10 min |
| 1. signs and measures of some emergencies: heart attack, stroke | 10 min | 10 min | / |
| 1. airway obstruction (child, adult) | 20 min | 10 min | 10 min |
| 1. other sudden illnesses:   complications of diabetes  epileptic seizure  anaphylactic reaction | 20 min | 15 min | 5 min |
| 1. falls and the most common injuries in the elderly population | 5 min | 5 min | / |
| 1. FA in the most common injuries of the elderly population: bleeding, broken arm, wounds, burns, hip fracture | 15 min | 5 min | 10 min |
| 1. course evaluation | 5 min |  |  |
|  | Total time: 2 h in 35 min |  |  |

**EXISTING FIRST AID COURSE PROGRAMME FOR THE GENERAL (LAY) POPULATION (for control group)**

| **TOPICS** | **TOTAL TIME** | **THEORY (minutes)** | **PRACTICAL EXERCISE (minutes)** |
| --- | --- | --- | --- |
| Introduction - general part:  - definition of FA,  - FA objectives,  - the moral and legal aspects of FA,  - triage,  - accident approach and procedures. | 30 min | 30 min | - |
| Injuries and FA:  - bleeding,  - wounds,  - burns,  - joints and bones injuries,  - shock. | 65 min | 20 min | 45 min |
| Basic resuscitation procedures and use of AEDs:  - emergency number and call  - unconsciousness and cardiac arrest,  - infant, child and adult CPR using AEDs,  - CPR of the drowned person,  - measures for airway obstruction with a foreign body. | 120 min | 30 min | 90 min |
| Dangerous sudden illnesses, poisoning, and FA:  - heart attack,  - stroke,  - epileptic seizure,  - complications of diabetes,  - acute poisoning,  - anaphylactic reaction. | 40 min | 40 min | - |
| Course evaluation:  – evaluation and completion of the course | 15 min | 15 min |  |
|  | Total time: 4 hours and 30 minutes |  |  |
