# Supplementary material for: Evaluation of a newly developed first aid training programme adapted for older people
Source: BMC Emerg Med. 2023 Nov 10;23:134. doi: 10.1186/s12873-023-00907-6 (PMC10636823; doi:10.1186/s12873-023-00907-6)
Supplement: Supplementary file 3 — Supplementary Material 3 [file 12873_2023_907_MOESM3_ESM.docx]

APPENDIX C

**THE PHYSICAL CAPABILITY TEST BATTERY**

The final number of points is the sum of the points collected from the following tests:

from Senior Fitness Test (Langhammer and Stanghelle, 2015)

1. BODY MASS INDEX (BMI)
2. THE CHAIR STAND TEST
3. THE BICEPS CURL TEST
4. FOUR SQUARE STEP TEST (Dite in Temple, 2002)
5. PICKING UP AN OBJECT FROM THE GROUND FROM A STANDING POSITION as BALANCE SCALE (Berg et al., 1989)

Instructions for performing the tests and reference values are given in:

Berg K, Wood-Dauphine S, Williams JI, et al. (1989) Measuring balance in the elderly: preliminary development of an instrument. *Physiotherapy Canada* 41: 304–11.

Dite W and Temple VA (2002) A clinical test of stepping and change of direction to identify multiple falling older adults. *Archives of physical medicine and rehabilitation* 83(11):1566–71

Langhammer B and Stanghelle JK (2015) The Senior Fitness Test. *Journal of physiotherapy* 61(3):163.

**PHYSICAL PERFORMANCE ASSESSMENT FORM**

| **RESPONDENT NUMBER** | | | | | | | |
| --- | --- | --- | --- | --- | --- | --- | --- |
|  | | | | | | | |
| **GENDER** | | | | | | | |
| M F | | | | | | | |
| **DATE OF BIRTH** | | | | | | | |
|  | | | | | | | |
| **HEIGHT** | cm | **WEIGHT** | kg | **BMI**  *weight x height²* | kg/m² | number of points: | 1 2 3 |

| **THE CHAIR STAND TEST** | | | | | | | | |
| --- | --- | --- | --- | --- | --- | --- | --- | --- |
| number of repetitions | |  | | | | | number of points:  1 2 3 | |
| **THE BICEPS CURL TEST** | | | | | | | | |
| number of repetitions (dominant upper limb) | | | | L D |  | | number of points:  1 2 3 | |
| **FOUR SQUARE STEP TEST (s)** | | | | | | | | |
| 1. | | 2. | | | | | number of points:  1 2 3 | |
| **PICKING UP AN OBJECT FROM THE GROUND FROM A STANDING POSITION (BALANCE SCALE)**  number of points: | | | | | | | | |
| 4 | 3 | | 2 | | | 1 | | 0 |
