# Supplementary material for: Evaluation of a newly developed first aid training programme adapted for older people
Source: BMC Emerg Med. 2023 Nov 10;23:134. doi: 10.1186/s12873-023-00907-6 (PMC10636823; doi:10.1186/s12873-023-00907-6)
Supplement: Supplementary file 4 — Supplementary Material 4 [file 12873_2023_907_MOESM4_ESM.docx]

APPENDIX D

**Please rate your knowledge of BASIC RESUSCITATION PROCEDURES (1 means very poor knowledge, 5 means excellent knowledge).**

1 2 3 4 5

**Please rate your knowledge of OTHER FIRST AID THEMES (not RESUSCIATION) (e.g. bleeding, care of injuries, measures in case of complications of diabetes, stroke, heart attack...) (1 means very poor knowledge, 5 means excellent knowledge).**

1 2 3 4 5

**FIRST AID THEORETICAL KNOWLEDGE TEST**

**1. Which of the following medical emergencies is most likely to occur in a person who is hungry, sluggish, sweaty, pale, shaky, and has a thinking disorder (difficulty concentrating, fatigue, confusion)?**

**2. Which of the following medical emergencies is most likely to occur in a person who speaks indistinctly, has a drooping corner of the mouth and a paralyzed half of the body?**

**3. Which of the following medical emergencies is most likely to occur in a person who has severe chest tightness and left arm pain, shortness of breath, nausea, and cold sweats?**

Possible answers to questions 1, 2, 3:

- stroke
- hypoglycaemia
- heart attack
- severe allergic reaction (anaphylaxis)

**4. What would you give to a person who is conscious and has complications of diabetes (hypoglycemia)?**

- chocolate
- water
- sugar (1 tablespoon)
- a cup of herbal tea
- nothing

**5. What would you do first if you recognized a conscious person with stroke in the evening time?**

**6. What would you do first if you recognized a conscious person with heart attack in the evening time?**

Possible answers to questions 5, 6:

- I would call 112 immediately
- I would wait until morning and take the person to the doctor myself
- I would start with cariopulmonary resusciation
- I would give a person a cup of herbal tea

**7. Which measure is most important if we find ourselves with a person who shows no signs of life?**

- chest massage with circular movements on the skin and compresses on the forehead
- stable position for the unconscious person
- rescue breaths
- use of a device Automatic external defibrillator
- strong and deep compressions on the middle of the chest

**8. What do we do if we find ourselves with a person who has broken his hip?**

- In any case, we call an ambulance
- We drive injured person to the nearest medical facility
- We drive injured to the nearest medical facility only if we do immobilization first
- We do nothing, because the hip can heal without the necessary interventions

**9. In which case would you use strong alcoholic drink (strong spirits) when providing first aid?**

- to disinfect wounds
- to reduce pain from injuries
- for stomach problems
- in no case

**10. In which case would you use home-made ointments when providing first aid?**

- for burn wounds
- in case of abrasions
- in no case
- in fractures
